# How the cell cycle impacts chromatin architecture and influences cell fate

**DOI:** 10.3389/fgene.2015.00019

**Published:** 2015-02-03

**Authors:** Yiqin Ma, Kiriaki Kanakousaki, Laura Buttitta

**Affiliations:** Department of Molecular, Cellular and Developmental Biology, University of Michigan, Ann Arbor, MI, USA

**Keywords:** cell cycle, chromatin, histones, nucleoporins, mitosis

## Abstract

Since the earliest observations of cells undergoing mitosis, it has been clear that there is an intimate relationship between the cell cycle and nuclear chromatin architecture. The nuclear envelope and chromatin undergo robust assembly and disassembly during the cell cycle, and transcriptional and post-transcriptional regulation of histone biogenesis and chromatin modification is controlled in a cell cycle-dependent manner. Chromatin binding proteins and chromatin modifications in turn influence the expression of critical cell cycle regulators, the accessibility of origins for DNA replication, DNA repair, and cell fate. In this review we aim to provide an integrated discussion of how the cell cycle machinery impacts nuclear architecture and vice-versa. We highlight recent advances in understanding cell cycle-dependent histone biogenesis and histone modification deposition, how cell cycle regulators control histone modifier activities, the contribution of chromatin modifications to origin firing for DNA replication, and newly identified roles for nucleoporins in regulating cell cycle gene expression, gene expression memory and differentiation. We close with a discussion of how cell cycle status may impact chromatin to influence cell fate decisions, under normal contexts of differentiation as well as in instances of cell fate reprogramming.

## INTRODUCTION

Chromatin serves as a platform for numerous cellular signals to influence gene expression. Post-translational modifications (PTMs) of histone proteins or covalent modifications of nucleotides influence a cell’s transcriptional program, which ultimately impacts cellular behavior and cell fate. Chromatin modifications are converted into transcriptional instructions by the interplay of modification “writers,” “erasers” and “readers” residing, often together, in a multitude of chromatin remodeling complexes that interact directly or indirectly with transcription factor complexes (Jenuwein and Allis, 2001). Like transcription factor complexes, the components of chromatin remodeling complexes may change with the differentiation status or fate of cells. For example lineage-specific chromatin remodeling complexes have been identified, as well as stem-cell specific complexes with functions in maintaining pluripotency (reviewed in Hargreaves and Crabtree, 2011).

Work by many groups over the past 10 years, including the extensive chromatin modification and accessibility mapping performed through the Encyclopedia of DNA Elements (ENCODE) and model systems-based ModENCODE projects have clarified that: chromatin accessibility and chromatin modifications are predictive of gene expression, DNA replication timing is correlated with an accessible chromatin structure, and chromatin is dynamic during fate acquisition and cellular reprogramming to pluripotency (for example, Ding and MacAlpine, 2011; Orkin and Hochedlinger, 2011; Thurman et al., 2012; Ho et al., 2014). However, with the exception of a few studies on replication timing, much of the mapping in these projects has used either asynchronously dividing cell lines, whole animals of various developmental stages, or tissues containing mixed cell lineages with differing cell cycle dynamics. How exactly the cell cycle status of a cell influences its chromatin state and how this impacts cell fate and cell fate plasticity remains a largely unaddressed question.

**FIGURE 1:**
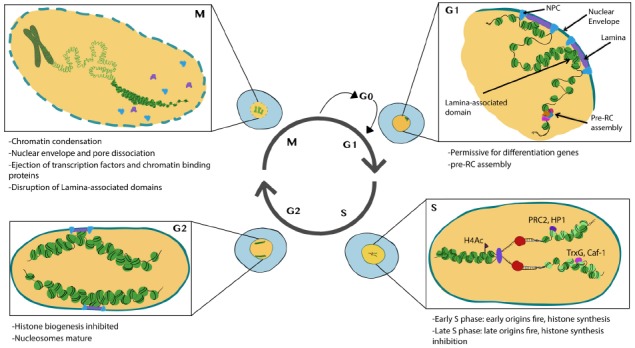
**Major features of chromatin and nuclear changes during the cell cycle.** Cells in G1 phase exhibit subnuclear domains with some regions associated with nuclear pores and nuclear lamina. Pre-RCs preferentially form at accessible chromatin. During S-phase histones are transcribed and synthesized, DNA is replicated and new (light green) and recycled (dark green) nucleosomes assemble to form nascent chromatin. PTM writers and readers also associate with nascent chromatin. During G2 nucleosomes mature and histone biogenesis is inhibited. During mitosis, chromosomes condense and many transcription factors and chromatin binding proteins are ejected from the chromatin. The nuclear envelope breaks down disrupting nuclear lamina associated domains. Illustration by Nicole Ethen.

Chromatin in proliferating cells is highly dynamic. Two important events occur during the cell cycle that allow for global chromatin restructuring. First, the incorporation of new histones onto nascent DNA occurs during S-phase and creates a requirement for the re-establishment of histone PTMs. Second, many chromatin remodeling complexes and transcriptional complexes are dissociated from chromatin during mitosis and the nuclear architecture, including chromatin domains or associations with the nuclear interior vs. periphery breaks down (Figure 1). This raises the question of how the cell maintains its transcriptional identity and fate through S-phase and mitosis. This question intersects with the field of *epigenetics*, which for the purposes of this review—is defined to encompass mechanisms that provide a cellular memory of gene expression, inheritable through the mitotic cell cycle (Berger et al., 2009). We define *cell fate* as a gene expression program that drives the acquisition of cell type-specific characteristics. Our goal in this review is to summarize recent findings that provide insight into how cell cycle status can influence chromatin and nuclear architecture to impact cell fate decisions. Also, we consider how developmental programs and acquisition of cell fate can feedback onto the expression of cell cycle regulators and cell cycle processes.

We begin our discussion with the regulation of histone biogenesis, key building blocks of chromatin. We then consider how the chromatin state influences the cell cycle through origin firing and chromosome compaction at mitosis. We focus on how the cell cycle impacts chromatin remodelers to coordinate these events and vice-versa. We then take a more global view of the nucleus, to discuss nuclear architecture and how nuclear domains and nuclear pore association impacts gene expression and DNA repair. These topics converge onto issues of how gene expression memory can be transmitted through the cell cycle and we discuss a central question in epigenetics; what are the epigenetic marks inherited through the cell cycle? Finally, we consider how the cell cycle status impacts chromatin to influence cell fate, in instances of cell fate acquisition and in the opposing direction of de-differentiation in nuclear reprogramming.

## CELL CYCLE DEPENDENT HISTONE BIOGENESIS

Histones are one of the primary components of chromatin and canonical histones (as opposed to histone variants) are actively synthesized during S-phase, in a manner coordinated with the replication of DNA. The speed of DNA replication is in fact tied to the rate of histone biosynthesis (Groth et al., 2007a; Gunesdogan et al., 2014; Mejlvang et al., 2014), suggesting new histone supply is tightly coupled to immediate demand during S-phase. The canonical histones consist of H1, H2A, H2B, H3, and H4 and they are small and highly positive charged proteins. Two copies of H2A, H2B, H3, and H4 form an octamer, which is wrapped by about 147 bp negative charged DNA (Richmond and Davey, 2003), resulting in the basic structure of the nucleosome. The canonical histone genes form clusters and present as one to several hundreds of copies depending on the species (Hentschel and Birnstiel, 1981; Marzluff et al., 2008). The transcription of histone gene takes place in a subnuclear organelle termed the histone locus body (HLB), containing factors required for the processing of histone pre-mRNAs which have an unusual mRNA structure, with a 3’UTR that forms a stem-loop structure instead of a polyA tail (White et al., 2007; Nizami et al., 2010). It has been suggested that excess free histones may be toxic to cells, explaining the evolutionary pressure for their conserved, yet peculiar regulation (De Koning et al., 2007).

**FIGURE 2:**
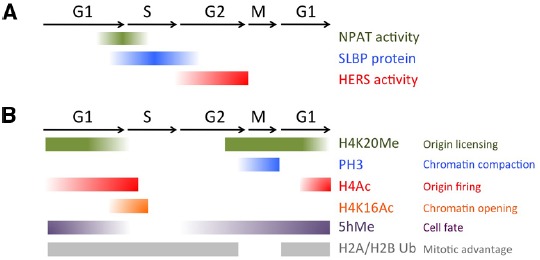
**Chromatin modifications and histone biogenesis regulators during the cell cycle. (A)** Factors controlling histone biogenesis are regulated by the cell cycle to limit histone biogenesis to S-phase. **(B)** Chromatin modifications, including histone PTMs and 5-hydroxy-methylcytosine (5hMe) occur in a cell cycle regulated manner to impact gene expression and nuclear architecture.

The onset and shut down of histone gene transcription is tightly regulated, in a manner elegantly coordinated with the core cell cycle machinery (De Koning et al., 2007; Groth et al., 2007b). Entry into S-phase is triggered by the activity of the G1-S Cyclin complex, CyclinE/Cdk2. In addition to phosphorylating targets to initiate DNA replication, CyclinE/Cdk2 also phosphorylates nuclear protein ataxia-telangiectasia locus (NPAT), to initiate transcription of the histone genes (Ma et al., 2000; Zhao et al., 2000; Ye et al., 2003). After CyclinE/Cdk2 activity has reached its peak in early S-phase, CyclinE/Cdk2 activity drops due to the degradation of the essential CyclinE component, thereby preventing further activation of NPAT until CyclinE re-accumulates in the next cell cycle (Figure 2).

While this simple mechanism could in theory be sufficient to limit histone biogenesis to S-phase, a direct regulator involved in robustly shutting down histone biogenesis after S-phase was also recently identified in *Drosophila*. The histone gene-specific epigenetic repressor in late S-phase (HERS) protein becomes phosphorylated by the late S-G2 Cyclin complex CyclinA/Cdk1, which localizes it to the histone genes where it acts to silence histone genes after S-phase (Ito et al., 2012). HERS silences histone gene expression by recruiting the repressive chromatin writer Su(var)3-9 for Histone H3 trimethylation at Lysine 9 (H3K9Me3), which subsequently recruits an H3K9Me3 “reader,” the transcriptional repressor Heterochromatin protein 1 (HP1). This recruitment of HP1 to the histone genes stably represses histone mRNA expression throughout G2 and early M. Importantly, the activity of the CyclinA/Cdk1 complex is kept low during G1 and early S-phases through the cell cycle-coupled degradation of CyclinA, triggered by the Anaphase Promoting Complex/Cyclosome (APC/C). This window of low CyclinA/Cdk1 during G1 allows cells to “reset” the inhibition of histone gene transcription and prepare for re-activation via the next pulse of CyclinE/Cdk2, to trigger NPAT activation (Figure 2).

In addition to the careful regulation of histone mRNA transcription, histone mRNA stability is also tightly regulated to limit transcript accumulation to S-phase. The conserved 3’ UTR of metazoan canonical histone transcripts forms a “stem-loop” structure, which binds stem-loop binding protein (SLBP). SLBP is involved in several aspects of histone mRNA metabolism, including histone pre-mRNA maturation, translation and degradation (Marzluff et al., 2008). Perhaps not surprisingly, the SLBP protein itself is cell cycle regulated. SLBP mRNA is synthesized constantly throughout the cell cycle, but SLBP becomes translated just prior to S-phase entry and the protein is degraded at the end of S- phase (Whitfield et al., 2000). SLBP protein stability is controlled by CyclinA/Cdk1, which phosphorylates a phosphodegron to trigger SLBP destruction (Zheng et al., 2003; Koseoglu et al., 2008). Altogether, both activation and repression of histone biosynthesis are very rapid, robust and directly coupled to the Cyclin/Cdk activity oscillations driving the cell cycle (Figure 2). This allows histone biogenesis to respond to all the cell fate cues that feed into regulating the speed and dynamics of the cell cycle during development, and under different signaling and environmental conditions.

## CHROMATIN ARCHITECTURE IMPACTS THE FORMATION OF ORIGINS FOR DNA REPLICATION

The DNA replication machinery is exquisitely regulated to ensure that the genomic DNA is copied only once within the cell cycle, with the interesting exception of highly specialized cells which re-replicate specific genomic regions to amplify certain genes (Nordman and Orr-Weaver, 2012). Replication is set up in three basic steps; first, the origin recognition complex (ORC complex) somehow identifies and binds to future origins on the chromatin just after mitosis and during early G1 (Mechali, 2010; Alabert and Groth, 2012). Next, during G1 the pre-replication complex (pre-RC) assembles on the ORC-bound locations. Pre-RC formation is marked by Cdt1 and Cdc6 recruitment of the minichromosome maintenance complex (MCM) complex. The successful assembly of a pre-RC then “licenses” origins for the third step, origin firing during S-phase. Firing is triggered in part by Dbf4/Cdc7 kinase (DDK) and CyclinE/Cdk2-dependent phosphorylations of origin complex components, leading to the recruitment of helicases and enzyme complexes for DNA replication (Zegerman and Diffley, 2007; Boos et al., 2013; Ramer et al., 2013).

A fundamental question about DNA replication is where on the genome replication starts. Unlike prokaryotes and yeast, metazoans have no obvious DNA sequence to designate origins of replication. Furthermore, there are estimated to be 30,000–50,000 potential origins of replication in the human genome, only about 10% of which are used within a given adult somatic cell cycle, suggesting most potential origins lie dormant (Alabert and Groth, 2012). This vast excess of origins may be important during rapid embryonic S-phases, and dormant origins can become activated when cells are placed under stress to avoid an S-phase delay (Courbet et al., 2008). It is widely believed that the choice of origins is developmentally controlled (Claycomb and Orr-Weaver, 2005) and consistent with this, different cell types exhibit distinct DNA replication patterns (Hansen et al., 2010).

Genome-wide analysis of DNA replication has expanded the numbers of predicted origins in *Drosophila*, mouse and human cells, and there is a strong correlation between origins and regions of active transcription (Cadoret et al., 2008; Sequeira-Mendes et al., 2009; Karnani et al., 2010; MacAlpine et al., 2010; Mesner et al., 2011). ORC binding, the first step in origin formation, is enriched in nucleosome-depleted regions suggesting DNA accessibility may be a major determinant in origin choice (MacAlpine et al., 2010; Lubelsky et al., 2014). However, not all open chromatin regions can serve as origins, indicating that origin specification involves additional factors yet to be determined. ORCs can also bind heterochromatin, though several additional factors are required to facilitate binding such as (HP1; Pak et al., 1997; Schwaiger et al., 2010; Cayrou et al., 2011), high mobility group protein HMGA1a (Thomae et al., 2008) and leucine-rich repeats and WD40 repeat domain-containing protein 1 (LRWD1) also known as ORCA (Shen et al., 2010). ORCs can also play origin-independent roles in generating repressive chromatin (Sasaki and Gilbert, 2007), therefore it has been challenging to tease out whether the recruitment and binding of ORC to heterochromatin functions in origin choice or serves other chromatin remodeling roles. In the cases of ORC recruitment by HMGA1a and ORCA, ORC recruitment does promote preRC formation and functional origins, suggesting these proteins facilitate ORC binding for origin formation in heterochromatin (Thomae et al., 2008; Shen et al., 2012).

While ORC binding may be rather widespread in the genome, this is only the first step in origin selection. The assembly of the pre-RC complex, the second step in origin formation, is also influenced by the chromatin state. Regions with high H4 acetylation are enriched for Pre-RC assembly during G1, and histone acetylation can promote origin licensing (Iizuka et al., 2006; Miotto and Struhl, 2008, 2010). The MYST-family histone acetyltransferase (HAT) HBO1 preferentially acetylates H4 on Lysines 5, 8, and 12 and is essential for proper DNA replication in human cells and *Xenopus* egg extracts (Doyon et al., 2006; Iizuka et al., 2006). An acetyltransferase defective HBO1 is unable to load MCMs for pre-RC formation, despite binding properly to origins (Miotto and Struhl, 2010). This suggests chromatin modifiers can specifically influence the step of replication licensing in G1. However, conspicuously, the loss of HBO1 in mice leads to decreased H3K14 acetylation, as opposed to H4 acetylation, and no obvious defects in DNA replication or cell cycle arrest were observed in HBO1 mutant embryos (Kueh et al., 2011). This unexpected finding suggests perhaps other MYST-family acetyltransferases can compensate for the absence of HBO1 *in vivo*, or possibly the role of HBO1 in preRC formation is more cell-type or context-dependent than thought.

Replication licensing also coincides with a specific histone PTM, monomethylation of H4 Lysine 20 (H4K20Me). H4K20Me levels fluctuate during the cell cycle, peaking during M and early G1 and plummeting during S phase (Tardat et al., 2010). The high levels of H4K20Me at mitosis suggest this mark could be involved in the earliest stage of origin choice (Figure 2). Indeed, artificially tethering the H4K20 methyltransferase PR-set7 to a non-origin chromatin region is sufficient to promote the ectopic loading of pre-RC components to that site. However, when PR-set7 is inhibited, loading of MCMs for licensing is impaired yet ORC binding to chromatin remains (Tardat et al., 2010). This suggests that H4K20Me may serve to reinforce origin licensing, perhaps acting sequentially in cooperation with HBO-dependent H4 acetylation.

## CHROMATIN AND THE TIMING OF ORIGIN FIRING

Not only is ORC binding and origin licensing impacted by the chromatin state, but origins are fired in a sequential way, such that some regions of the genome replicate early while others replicate late in S phase (Mechali, 2010). Such differential timing in origin firing is highly conserved from fission yeast to humans, and whether this has some evolutionary advantage or is simply a consequence of complex nuclear architecture remains unclear. The timing of origin firing is dynamic during development and different between cell types (Hansen et al., 2010; Farrell et al., 2012). Perhaps not surprisingly, the timing of origin firing correlates with the data on sites of Pre-RC assembly at late M- early G1. Early replicating regions are commonly enriched in H4 acetylation and are associated with actively transcribed, accessible chromatin (Kemp et al., 2005; Goren et al., 2008; Schwaiger et al., 2009; Hansen et al., 2010; Lubelsky et al., 2014). In cells treated with histone deacetylase (HDAC) inhibitors, late replicating origins can shift toward earlier replication (Kemp et al., 2005; Goren et al., 2008) suggesting that opening chromatin has functional consequences on origin firing.

A direct relationship between origin firing and H4 acetylation was reported in yeast (Vogelauer et al., 2002) and *Xenopus* (Danis et al., 2004), and was carefully dissected in a study of specialized origins located near the chorion genes in the follicle cells of the *Drosophila* ovary (Aggarwal and Calvi, 2004). The follicle cells are tasked with quickly producing and secreting the eggshell (chorion) for the developing egg in the ovary. In order to accomplish this, the follicle cells amplify the copy numbers in the regions of the genome encoding the chorion genes by repeatedly re-firing origins at a specific stage of development in the ovary (Nordman and Orr-Weaver, 2012). Thus, the level of chorion gene amplification can serve as a read-out for the firing rate of an isolated origin. This unique feature of origin re-firing and re-replication has allowed for detailed *in vivo* genetic analyses of origin firing, unparalleled in any other system.

Acetylation of H4, in particular acetylation at H4K8, directly correlates with the levels of chorion gene amplification and thus origin re-firing (Kim et al., 2011). When the HDAC Rpd3 is tethered to a chorion amplification origin, amplification and origin re-firing becomes hindered (Aggarwal and Calvi, 2004). By contrast, recruitment of the acetyltransferases CREB-binding protein (CBP) and HBO1 to licensed amplification origins promotes re-firing (McConnell et al., 2012). H4 acetylation could promote origin firing through increasing the accessibility of DNA to the helicase complexes needed for replication fork movement, or by facilitating histone octamer eviction for DNA unwinding via the remodeling SWI/SNF and RSC complexes (Ferreira et al., 2007). These models suggest a passive role for the chromatin state in regulating origin firing though, by simply limiting the access or movement of replication enzymes. It would be interesting to examine whether H4 acetylation may also impact or regulate the ability of CyclinE/Cdk2 to phosphorylate its substrates at licensed origins to initiate firing.

In contrast to early replicating origins and origins for gene amplification, late-firing origins are usually associated with a repressive, closed chromatin structure. For example HP1-bound regions near centromeric heterochromatin repeats in *Drosophila* replicate late, and reducing HP1 levels leads to earlier replication of these centromeric repeats (Schwaiger et al., 2010). The later replication of heterochromatin could be due to a reduced density of ORC bound regions, reduced pre-RC formation, or chromatin that is simply less accessible to helicases and replication enzymes. However, it is worth noting that a subset of heterochromatin replicates early in *Drosophila* and fission yeast (Hayashi et al., 2009; Schwaiger et al., 2010; Cayrou et al., 2011). In these cases, paradoxically the HP1/ORC association promotes ORC recruitment and earlier origin firing. Such differential roles for HP1 in heterochromatin replication imply that a compact chromatin structure is not the only factor dictating replication timing, and beg the question of what other factors can influence the timing of origin firing.

Recent work in early *Drosophila* embryos has investigated the initial formation of late- replicating heterochromatin in detail. The earliest appearance of late-firing origins in *Drosophila* embryos occurs at repetitive satellite DNA during the mid-blastula transition when zygotic transcription is first activated (Shermoen et al., 2010). Farrell et al. (2012) recently discovered that providing a low level pulse of early Cdk1 activity can push the very first late-firing origins in *Drosophila* development to replicate early. This finding is surprising for two reasons. First Cdk1 activity is normally associated with triggering mitosis and preventing re-licensing of replication origins, so a role for Cdk1 in promoting origin firing is unexpected. Second, Farrell et al. (2012) found that Cdk1 can promote the earlier firing of late origins even at a time when these regions of the genome already exhibit a more compacted chromatin structure (Shermoen et al., 2010; Farrell et al., 2012). This suggests that perhaps local Cyclin/Cdk activity may somehow be able to overcome a compacted chromatin structure to influence the timing of origin firing when needed in specific contexts.

Most likely, both local Cyclin/Cdk activity and chromatin structure ultimately impact the timing of origin firing. Importantly, the initial formation of late-firing origins does require activation of the zygotic transcription program (Shermoen et al., 2010) which underscores the close relationship between gene expression, chromatin accessibility and timing of origin firing during development. Methods to examine the 3D structure and organization of chromatin in the nucleus such as Chromatin Conformation Capture, termed “3C” or “Hi-C,” have established that different mammalian cell types contain topologically associated chromatin domains or “TADs,” thought to be the results of cell-type specific chromatin sub-compartments (Dixon et al., 2012). Recent work from the Gilbert lab has revealed that TADs also share replication timing features, further demonstrating in mammalian cells that cell-type specific nuclear architecture correlates with replication timing (Pope et al., 2014). Their model, derived from analysis of over 30 mouse and human cell types, suggests DNA replication initiates within TADs permissive for transcription but replication forks gradually advance later into TADs that are repressive for transcription. Importantly, whether transcription establishes the nuclear architecture that influences replication timing, or whether replication timing somehow establishes the nuclear subdomains that impact transcription remains unresolved. Since gene expression and nuclear architecture differs between cell types and changes with the acquisition of cell fate (Peric-Hupkes et al., 2010), it is likely that origin usage and the timing of origin firing will be equally as dynamic during development as gene expression.

## WHAT ARE THE EPIGENETIC MARKS?

A qualified epigenetic mark should be faithfully transmitted to daughter cells through DNA replication and cell division. Nucleosomes and the associated chromatin architecture must dis-assemble before replication forks and re-assemble with newly synthesized DNA and histones after forks pass (Margueron and Reinberg, 2010). This poses a challenge for cells to maintain their non-DNA sequence information, such as DNA methylation and histone modifications. The semi-conservative mechanism of DNA synthesis is thought to provide an effective way to ensure the inheritance of DNA methylation through hemi-methylation dependent maintenance methylases such as the cytosine methyltransferase Dnmt1 in mammals (reviewed in Law and Jacobsen, 2010). Dnmt1 is recruited to nascent chromatin by Ubiquitin-like PHD and RING finger domain 1 protein (UHRF1), which recognizes hemimethylated CG dinucleotides (Bostick et al., 2007; Sharif et al., 2007). Dnmt1 can also interact with a component of the moving replication fork, proliferating cell nuclear antigen (PCNA; Chuang et al., 1997), to promote cytosine methylation immediately after new DNA synthesis. However, some common genetic model organisms lack substantial genomic cytosine methylation, such as budding yeast, *C. elegans* and *Drosophila* (Proffitt et al., 1984; Simpson et al., 1986; Takayama et al., 2014), demonstrating that DNA methylation is not a universal epigenetic mark.

The case of inheriting histone modifications seems more challenging. There is no obvious nucleosome template to directly copy and newly synthesized, unmodified histones are incorporated into the nascent DNA (Probst et al., 2009). A model has been suggested for the inheritance of the H3K27Me3 modification through the cell cycle, based on the observation that this modification can directly recruit a complex containing both PTM writing and binding activity, the PRC2 complex (Hansen et al., 2008). PRC2 contains the H3K27Me3 writer, *Enhancer of zeste* (or EZH2 in humans), as well as an H3K27Me3 binding subunit *Extra sexcombs*, (or EED in humans). Importantly, EED binding to the H3K27Me3 modification stimulates the methyltransferase activity of EZH2, thereby providing an intuitive way for the PRC2 complex to propagate the H3K27Me3 modification (Margueron et al., 2009). The model posits that the PRC2 complex is recruited to chromatin by the H3K27Me3 modification in G1, and enough PRC2 is recruited to H3K27Me3 on mature histones that are recycled and re-incorporated into the replicated DNA during S-phase to allow for H3K27 modification on nearby, newly incorporated histones (Hansen et al., 2008; Margueron and Reinberg, 2010). Such a mechanism is not necessarily H3K27 specific, and could be shared with other histone PTMs. For example, H3K9 is di- or tri-methylated by Su(var)3-9, which is read by the chromodomain of HP1. HP1 then further recruits Su(var)3-9, thereby leading to the spreading, or potentially also the maintenance, of H3K9 methylation on new histones (Bannister et al., 2001; Lachner et al., 2001). Similar interactions could also exist between histone acetylation and HATs, which are often located in complexes that contain acetyl-histone readers, such as bromodomain proteins (Dhalluin et al., 1999; Filippakopoulos et al., 2012; Filippakopoulos and Knapp, 2014). Future studies on the association of additional PTM writer/reader complexes with nascent DNA through the cell cycle may support a similar model for propagation of multiple histone PTMs during DNA replication.

Such a model creates a “chicken and egg” type-conundrum though when asking what is the inherited epigenetic mark in dividing cells, as it seems to be both the histone PTM itself and the writer/reader complex. Indeed, recent work in human cell lines seems to support this model. Alabert et al. (2014) isolated newly replicated chromatin to profile the association dynamics of thousands of chromatin binding proteins and to compare the levels of histone PTMs in nascent chromatin versus mature chromatin. They found that specific histone PTMs such as H3K27Me3 and H3K9Me3 remained similar between nascent and “mature” chromatin, and when the synthesis of new histones is blocked, H3K27Me3 and H3K9Me3 remain abundant on nascent chromatin. This implies that significant amounts of certain PTMs on nascent chromatin can originate from the old recycled histones (Alabert et al., 2014). In further support of the model, they also find the PRC2 complex is present in both nascent and mature chromatin, consistent with rapid recruitment by recycled parental histones carrying H3K27Me3.

However, a very different model for inheritance of the epigenetic mark through S-phase was proposed by a study of early stage *Drosophila* embryos (Petruk et al., 2012). Petruk et al. (2012) found that the H3K27Me3 mark is actually very low during S-phase in cells of the *Drosophila* gastrula and is not detectable on the newly synthesized DNA until later in G2 phase. They reasoned that the true epigenetic modifications should be re-established shortly after DNA replication. To determine which PTMs or chromosomal proteins are in close proximity to the replication machinery, they used a “proximity ligation assay” (PLA) approach. In this assay, proteins or histone PTMs that are within 30–40 nm of replication forks containing PCNA generate a fluorescent signal, with a sensitivity that allows visualization of single molecule interactions *in vivo* (Soderberg et al., 2006). In the *Drosophila* embryo, several histone modification writers and readers including E(z), TrxG, Pc, Caf-1, LID, UTX, and HP1 are tightly associated with the replication forks, and are located on nascent DNA during S phase. However, their corresponding histone PTMs were not associated with replication forks, nor detectable on nascent DNA until ∼1 hr after the passage of replication fork, which is already G2 phase at this stage of development. This suggests that it is the PTM writers that remain associated with nascent chromatin during replication which must act to re-establish PTMs later. Thus, it seems the chromatin binding of the PTM writers rather than the PTMs themselves may serve as a true, inherited epigenetic mark. Although surprising, this work is consistent with a previous study showing that Polycomb remains bound to replicating chromatin *in vitro* (Francis et al., 2009). The methyltransferase SET domain of PTM writers can bind single-stranded DNA *in vitro*, suggesting a manner in which they may be retained on newly synthesized DNA independent of a recruiting PTM (Krajewski et al., 2005). Self-association and oligomerization may be another manner in which PTM writers can be maintained in the absence of a recruiting PTM (Lo et al., 2012) and finally, Polycomb complexes can be recruited to DNA in a sequence-specific manner through Polycomb response elements or PREs, which recruit complexes during early S-phase prior to replication (Lanzuolo et al., 2011). However, it remains unclear in the *Drosophila* embryo whether the PTM writers remain associated with the same specific locations on DNA before and after replication fork passage.

These seemingly conflicting observations of Alabert et al. (2014) and Petruk et al. (2012) are likely due to the developmental stage and cell cycle speed of the model systems under study. For example, in the *Drosophila* embryo it seems relatively few PTMs may have already been established on the mature nucleosomes at the stage of development under study. Indeed the authors show there is little to no H3K27Me3 at the cellular blastoderm stage before gastrulation. Thus perhaps when there are lower levels of established PTMs, they can be preceded by the binding of the histone modifiers in S-phase (Petruk et al., 2012). In contrast, the adult human cells have already heavily established PTMs in the chromatin prior to passage of the replication fork, and thus recycling histones containing PTMs allows them to more readily be used as a template to recruit modifying enzymes and re-establish the necessary chromatin modifications.

A new study using early *C. elegans* embryos throws yet another wrinkle into this epigenetic inheritance problem though (Gaydos et al., 2014). In contrast to the results in *Drosophila*, Gaydos et al. (2014) find that chromatin containing the H3K27Me3 PTM in *C. elegans* retains the mark through several early embryonic cell divisions, even in embryos lacking the H3K27Me3 writer enzyme. A chromosome inherited with the H3K27Me3 mark already established, retains it during early embryonic divisions exhibiting only the expected level of passive dilution due to new histone incorporation. While chromosomes in the exact same embryo- inherited without the H3K27Me3 mark already established, cannot establish it *de novo* until later in development. Thus, it seems clear the H3K27Me3 PTM itself in *C. elegans* embryos serves as an inherited epigenetic mark. Taken together, the studies of Petruk et al. (2012) and Gaydos et al. (2014) suggest there may be different modes of epigenetic inheritance used in different organisms. Perhaps flies use chromatin-bound PTM writers to carry the epigenetic information through early embryonic cell divisions, while worms use the PTM itself? An organism specific answer to the epigenetic inheritance question seems a bit unsatisfying, especially as all the ingredients, the PTMs, the readers, the writers and the S-phase machinery are so well conserved. Hopefully future studies will be able to reveal an underlying unifying concept to explain what is the true inherited epigenetic mark.

## CHROMATIN AND CHROMOSOME COMPACTION DURING MITOSIS

To ensure the fidelity of separating identical genetic information into two daughter cells, chromatin undergoes dramatic compaction during the cell cycle into mitotic chromosomes. Mitotic chromosomes are easily recognizable based on their morphology, however, the details of their three-dimensional structure have remained enigmatic. Recent use of advanced Chromosome Conformation Capture methods such as 5C and Hi-C in human cell lines performed at timepoints across the cell cycle, have revealed that mitotic chromosomes exhibit a common structure shared in multiple cell types (Naumova et al., 2013). Mitotic chromosomes appear to be organized as a linear array of chromatin loops of variable size, which are then tightly compressed together longitudinally. The common structure of mitotic chromosomes seems striking, given the cell type-specific subdomains and features of interphase chromatin structure, such as TADs (Pope et al., 2014). This suggests that some cell-type specific chromatin architecture is lost during mitosis and higher-order chromatin structures form *de novo* after mitosis.

Accompanying this dramatic chromatin compaction is the alteration of chromatin-based activities, such as the cessation of transcription (Martinez-Balbas et al., 1995; Gottesfeld and Forbes, 1997). This is thought to be accomplished in part, by the inhibition of transcription factor binding to the mitotic chromatin. For example, the large C2H2 zinc finger transcription factor family becomes phosphorylated at the conserved linker region during mitosis, which leads to dissociation from mitotic chromatin (Dovat et al., 2002; Rizkallah et al., 2011). Alternatively for specific transcription factors that remain bound to the mitotic chromosome, such as FoxA1 and GATA1, their co-activators can be excluded from mitotic chromatin. This mechanism may allow the transcription factors to act as platforms for timely reactivation of transcription after mitosis, a mechanism termed “mitotic bookmarking” which has been discussed in detail elsewhere (Kadauke et al., 2012; Caravaca et al., 2013; Kadauke and Blobel, 2013; Wang and Higgins, 2013).

DNase sensitivity has been used to probe chromatin accessibility during different stages of the cell cycle. Somewhat surprisingly and in contrast to the Hi-C data mentioned previously, DNase sensitivity is widely preserved from interphase to mitosis (Hsiung et al., 2014). During interphase, DNAse sensitivity generally corresponds to transcription factor binding sites and active gene proximal promoters. While in mitosis, gene expression ceases, higher order chromatin domains are lost and many transcription factors are ejected. So why and how are most DNase sensitive regions maintained during mitosis? First to be precise, there are a few expected alterations to accessibility in mitosis. For example, distal regulatory elements that bind transcription factors are somewhat more likely to lose accessibility during mitosis compared to gene proximal promoters. Second, chromatin modifications and some chromatin modifiers are retained on the mitotic chromosomes and can help to preserve local chromatin structure, even if higher order structures are disrupted, as suggested by the Hi-C data. For example, the trithorax protein MLL maintains its chromatin association during mitosis, and loss of MLL impairs the rapid reactivation of MLL target genes after mitotic exit (Blobel et al., 2009). This process is reminiscent of the mitotic bookmarking described above, and suggests that retention of a few key chromatin modifiers during mitosis may be all that is needed to transmit gene expression information and maintain cell fate through mitosis.

What are the histone PTMs involved in compacting the chromatin at mitosis? The best-documented mitotic chromatin mark is phosphorylation of the H3 N-terminal tails. Four major residues of H3 are phosphorylated during mitosis, T3, S10, T11, S28, in a manner conserved from yeasts to humans (Rossetto et al., 2012). Aurora B is the major kinase responsible for these phosphorylations, which can be counteracted by the Protein Phosphatase 1 (PP1). Insufficient H3 phosphorylation leads to abnormal chromosome condensation and segregation, which is due to impaired recruitment of Condensin I complexes (Adams et al., 2001; Giet and Glover, 2001). The Condensin complex is the major effector of chromosome condensation during mitosis. In the presence of type I topoisomerases, Condensins progressively wind and fold the chromatin fiber into supercoils, which compact to form the mitotic chromosome (Hirano, 2012; Thadani et al., 2012; Aragon et al., 2013). Importantly though, phosphorylation of H3 does more than simply recruit Condensins, it can also modulate the binding of repressive chromatin proteins to mitotic chromosomes. For example, H3K9 the residue adjacent to H3S10 can be methylated and its trimethylation recruits the HP1 reader to form heterochromatin. However, during mitosis the majority of HP1 is released from chromatin, due to phosphorylation on H3S10, which ejects HP1 from binding H3K9Me3 on mitotic chromatin (Fischle et al., 2005). Something similar may also occur with H3K27, which recruits the Polycomb complexes PRC1 when methylated and lies adjacent to the H3S28 phosphosite (Wang and Higgins, 2013).

H4K20 mono-methylation (H4K20Me), the same PTM mentioned earlier to promote pre-RC formation, is also required for proper chromosome condensation (Karachentsev et al., 2005; Sakaguchi and Steward, 2007; Houston et al., 2008; Oda et al., 2009). H4K20me facilitates chromatin condensation in part by antagonizing a second PTM, H4K16 acetylation (H4K16Ac; Nishioka et al., 2002). H4K16Ac inhibits chromatin compaction, and consistent with a role in opening chromatin, its levels normally peak during S phase (Shogren-Knaak et al., 2006) and decrease during mitosis (Rice et al., 2002; Figure 2). H4K20Me is also thought to contribute to chromosome compaction in early M phase by binding specific components of the Condensin II complex (Liu et al., 2010). Condensin II binds to interphase chromatin and is thought to mediate early phases of chromatin compaction, well before Condensin I. Altogether this suggests a two-step model for chromatin modifications to promote chromosome compaction at mitosis. First, H4K20Me limits H4 acetylation and recruits Condensin II. This then cooperates with Aurora B triggered H3 phosphorylation to eject H3K9-and possibly H3K27 -bound protein complexes and recruit Condensin I during early metaphase for further compaction (Ono et al., 2003). In this manner, the compaction of the chromatin at mitosis and the ejection of certain chromatin bound factors are directly coupled.

## REGULATION OF HISTONE MODIFIERS BY THE CELL CYCLE MACHINERY

While chromatin impacts cell cycle events like origin firing and chromosome segregation at mitosis, the cell cycle machinery also impacts chromatin by regulating the histone modifiers. The activity of certain histone modifiers fluctuates in a cell cycle-dependent manner. Perhaps the best-studied example of this is the regulation of the H4K20 mono-methyltransferase PR-Set7 and its opposing de-methylase, PHF8 (Rice et al., 2002; Liu et al., 2010). Both PR-Set7 mRNA and protein levels peak during G2 and mitosis, only to plummet during G1, consistent with the observed changes of the H4K20Me PTM (Rice et al., 2002). The dynamic regulation of PR-Set7 is in part due to its proteolytic degradation during S-phase. PR-Set7 contains a conserved PCNA-interacting peptide (PIP-box) which mediates its association with the PCNA component of the replication fork. The binding to PCNA during S-phase is recognized by the E3 ubiquitin ligase CRL4/Cdt2, which leads to degradation of PR-Set7 and PCNA, in order to prevent pre-mature chromatin compaction prior to M-phase (Abbas et al., 2010; Centore et al., 2010; Oda et al., 2010). Conversely, the PHF8 de-methylase becomes phosphorylated by the mitotic Cyclin complex, CycB/Cdk1, resulting in its dissociation from mitotic chromosomes to allow for the accumulation of H4K20Me and subsequent recruitment of Condensin II (Liu et al., 2010).

In addition to H4K20 associated modifiers, cell cycle dependent regulation of other PTM writers has also been reported. EZH2, the mammalian homolog of Enhancer of zeste, E(z) in *Drosophila*, is the major methyltransferase for H3 Lysine 27 and plays a crucial role in differentiation gene silencing through interaction with the Polycomb Repressive Complex 2 (PRC2; Cao et al., 2002; Kuzmichev et al., 2002; Muller et al., 2002). EZH2 is a direct target of the core cell cycle transcriptional regulator E2F (Bracken et al., 2003), and is up-regulated in proliferating stem cells or cancer stem cells, where it has been suggested to maintain pluripotency (Varambally et al., 2002; Lee et al., 2006; Sparmann and van Lohuizen, 2006; Simon and Lange, 2008). Several groups also uncovered a direct link between EZH2 and Cyclin/Cdks. The key S-phase and M-phase kinases, CDK1 and CDK2 can phosphorylate EZH2 in a cell cycle dependent manner on Thr350. This phosphorylation reinforces differentiation-associated gene silencing, such as silencing of HOX genes and SOX family members, and is thought to maintain stem cell identity (Chen et al., 2010; Kaneko et al., 2010). However, EZH2 can also be phosphorylated by CDK1 at Thr487, which disrupts the binding of EZH2 to the other PRC2 components, leading to the de-repression of EZH2 target genes, resulting in premature osteogenic differentiation of human mesenchymal stem cells (Wei et al., 2011). Thus, the cell cycle regulation of EZH2 can have both positive and negative outcomes on stem cell identity and differentiation. How these outcomes are balanced in actively proliferating cells remains unclear. Although there is plentiful data suggesting that EZH2 is important for normal cell proliferation and maintaining stem cell identity, whether part or all of these functions occur through PRC2-dependent gene silencing or another role of EZH2 is not known. PRC2-independent roles for EZH2 have been described, including an unexpected function as a transcriptional co-activator (LaJeunesse and Shearn, 1996; Strutt et al., 1997; Lee et al., 2011; Xu et al., 2012). To fully understand how EZH2 coordinates with the cell cycle machinery to promote proliferation and maintain stem cell identity, further investigations will be required.

These specific examples of the cell cycle machinery impacting chromatin modifiers are likely to be only the tip of the iceberg. The Cyclin/Cdk complexes themselves have hundreds of targets, many of which are uncharacterized or remain to be identified (Ubersax et al., 2003; Chi et al., 2008). In addition the myriad of other cell cycle kinases, phosphatases, ubiquitin ligases and their targets are only recently being uncovered on a proteomic scale (Bernal et al., 2014; Kuilman et al., 2014; Li et al., 2014; Lipinszki et al., 2014). Such large-scale approaches are likely to reveal new connections between the cell cycle machinery and chromatin regulators, which lie at the core of coordinating gene expression, with genome duplication and segregation.

## GLOBAL NUCLEAR ARCHITECTURE AND THE CELL CYCLE: THE INTERACTION OF CHROMATIN WITH THE NUCLEAR ENVELOPE

Chromatin is not organized randomly within the nucleus during interphase, and microscopic observations of mammalian nuclei revealed that condensed chromatin is localized preferentially in the nuclear periphery, interrupted by stretches of less condensed chromatin at the nuclear pore complexes (NPCs). This distribution of heterochromatin-euchromatin led to the hypothesis that the more open chromatin near nuclear pores represents actively transcribed regions, and that this interaction facilitates the coupling of transcription with mRNA export, a process termed “gene gating” (Blobel, 1985). Consistent with this idea, active genes in yeast have been found to be localized at the Nuclear pore basket, including housekeeping genes and inducible genes that become re-located to the NPCs upon activation (Dieppois and Stutz, 2010; Burns and Wente, 2014). The recruitment of active genes to the NPCs in yeast involves interactions between the Nuclear Basket Nucleoporins or Nups (Mlp1, Nup1) with a HAT complex SAGA, and the TRanscription-EXport complex TREX-2 (Cabal et al., 2006; Luthra et al., 2007). Gene recruitment to these regions is dependent upon specific sequences termed GRS I and II present in the inducible gene promoters (Ahmed et al., 2010).

In higher eukaryotes, the relationship of gene activation and Nuclear Pore binding is complicated due to the recent discovery that several Nups have “off-pore” roles in the nucleoplasm (Capelson et al., 2010; Kalverda et al., 2010; Liang et al., 2013; Buchwalter et al., 2014). In the special, amplified polytene chromosomes of *Drosophila* salivary glands, Nup98 and Nup50 can be observed bound to decondensed chromatin and sites of active transcription microscopically. Nup98 and another Nup, Sec13, are localized to transcribed genes prior to the initiation of transcription, and an RNAi knockdown of Sec13 or Nup98 reduces transcription and RNA polymerase II recruitment, demonstrating functional roles for this binding (Capelson et al., 2010; Kalverda et al., 2010). However, the same Nups can also bind different set of genes when located in the pore vs. nucleoplasm. Recent examination of Nup98 mutant forms that are either solely nucleoplasmic or NPC-tethered showed nucleoplasmic Nup98 binding to genomic regions with high gene expression, marked with Histone PTMs associated with open chromatin (H3K4Me2 and H4K16Ac). In contrast, NPC-tethered Nup98 bound genomic regions with average gene expression, that are low in Histone PTMs associated with transcription (Kalverda and Fornerod, 2010; Kalverda et al., 2010), a finding seemingly opposite to the gene-gating model in yeast. Thus, in metazoans actively transcribed genes bound by Nups are more likely to be found in the nucleoplasm while NPC binding is correlated with lower gene expression levels.

“Transcriptional gene memory” is an interesting case where Nucleoporin binding is associated with future gene re-activation rather than current expression levels. Transcriptional memory is a phenomenon whereby a recently expressed and shut-off gene is transcriptionally re-activated faster after exposure to the same stimulus for second time, allowing cells to respond quickly to environmental changes. This phenomenon can last through several cell divisions, demonstrating epigenetic inheritance (Brickner, 2009). In yeast, transcriptional memory of the INO1 gene requires a memory recruitment sequence (MRS) sequence in the promoter, incorporation of the H2A variant histone H2Az, and interaction of the promoter with the NPCs (Light et al., 2010). Transcriptional memory is conserved in mammals and also requires Nucleoporin binding. In HeLa cells the HLA-DRA gene induced by Interferon gamma (IFN-γ) exhibits transcriptional memory (Gialitakis et al., 2010), which is inherited through multiple cell divisions and is dependent upon the nucleoporin Nup98 (Light et al., 2013). However, as mentioned previously Nup98 can have both NPC and “off-pore” roles in metazoans, and importantly, the Nup98 interaction with the HLA-DRA promoter in human cells takes place in the nucleoplasm, not at NPCs (Light et al., 2013). In both cases, at yeast and human genes, transcriptional memory is associated with increased dimethylation of H3K4 (H3K4Me2) in the promoters, a mark which is dependent upon the interaction with the Nups (Light et al., 2013). However, H3K4 methylation is apparently not necessary for transcriptional memory, as deletion of the responsible Set1 methylase in yeast does not prevent transcriptional memory at Gal1 and Gal10 loci (Kundu et al., 2007; Laine et al., 2009). Overall, yeast and mammalian cells seem to share a common mechanism regarding transcriptional memory, which requires Nucleoporin binding, but in yeast this interaction occurs at the NPCs, while in mammals it occurs in the nucleoplasm. This distinction may be due to the “closed” nature of mitosis in yeast, where the nuclear envelope does not break down and is therefore is able to carry transcriptional memory through mitosis. In contrast the “open mitosis” of mammals may not be able to maintain transcriptional memory through M-phase and therefore this function has shifted to Nups located in the cytoplasm.

Outside of “gene gating” and transcriptional memory, chromatin binding to NPCs can also be associated with gene repression and silencing. In yeast the nucleoporin Nup170 interacts with the Sir4 subunit of the Silencing InsulatoR (SIR) complex, required for silencing of subtelomeres (Van de Vosse et al., 2013). The mammalian ortholog of Nup170 (Nup155) interacts with the HDAC4, also involved in transcriptional repression, revealing a conserved Nucleoporin function in silencing (Kehat et al., 2011). Because condensed chromatin is often found in the nuclear periphery between NPCs, yet many Nucleoporins are associated with actively transcribed genes, it has been suggested that specific Nups could create “transition zones” between heterochromatin and euchromatin (Van de Vosse et al., 2013), potentially reconciling the seemingly contradictory associations of Nups.

The localization of chromatin to the nuclear periphery, away from pores is suggested to be transcriptionally repressive in yeast and mammals (Andrulis et al., 1998; Malhas et al., 2007). Using this mechanism to silence gene expression involves chromatin movement from the nucleoplasm to the nuclear periphery. Chromosomes maintain certain positions in interphase nuclei (Chubb et al., 2002), and movement of artificial transgenes to the nuclear periphery in mammalian cells has been shown to require cell cycle progression through mitosis (Finlan et al., 2008; Reddy et al., 2008). This may be because the nuclear envelope-chromatin interactions need to be disrupted and re-established, an event driven by the open mitosis in mammalian cells. Importantly, this also suggests post-mitotic cells can use this repressive mechanism to permanently silence genes, and suggests a manner by which forcing cell cycle re-entry of postmitotic cells may promote chromatin re-localization and create a state permissive for cell de-differentiation (Nicolay et al., 2010; Pajcini et al., 2010).

Heterochromatin tethering along the nuclear periphery is mediated by lamins, nuclear cytoskeleton filaments, that connect chromatin to the inner nuclear membrane of the nuclear envelope (Dechat et al., 2008). Lamin-associated aomains (LADs) of the mammalian genome contain a relatively low number of genes and exhibit a repressed chromatin state (Guelen et al., 2008; Peric-Hupkes et al., 2010). LADs have been shown in a number of studies to modulate gene expression, and repositioning genes to a LAD is sufficient to mediate repression (Kosak et al., 2002; Williams et al., 2006; Reddy et al., 2008). One persistent question in the field though, has been how the chromatin associated with LADs can be “remembered” after nuclear envelope breakdown and reformation following mitosis.

A detailed analysis of LAD positioning during the cell cycle was performed using a modified Dam-ID approach, to permanently mark chromatin regions that associate with nuclear lamina, and track their position even after detachment and through the cell cycle (Kind et al., 2013). The study revealed that in a human cell line, LADs are generally found in nuclear periphery during interphase and are enriched for the H3K9Me2 PTM, associated with gene silencing. Interestingly, during mitosis the LADs remain distinct from regions of PTMs associated with transcriptional activity such as H3K27Ac and H3K4me2. However, after mitosis the LADs from the prior interphase do not re-establish a peripheral localization in the nucleus, instead they become distributed stochastically between the nucleoplasm and nuclear periphery. These results suggest that LAD positioning and the PTMs associated with it, are in fact, not mitotically inherited (Kind et al., 2013).

This profound and surprising result raises the question of how such stochastic changes in chromatin dynamics during each cell cycle, and presumably gene expression, can possibly be reconciled with seemingly organized and predictable changes in cell fate during development. One possibility is that LADs may be primarily used to modulate gene expression in postmitotic cells, although studies performed in proliferating fibroblasts suggest this may not be the case (Reddy et al., 2008). Importantly, new single-cell based assays are revealing a surprising amount of stochastic variation in individual cell decisions of quiescence vs. proliferation or differentiation vs. pluripotency, even within clonal cell populations in culture (Kalmar et al., 2009; Dey-Guha et al., 2011; Spencer et al., 2013). Does the inherent unpredictability of chromatin reorganization after mitosis possibly underlie this stochasticity? This will be an interesting question to address in future research.

## GLOBAL NUCLEAR ARCHITECTURE AND THE CELL CYCLE: OPEN MITOSIS AND THE NUCLEAR PORE COMPLEX

In metazoan cells where an “open mitosis” takes place, the nuclear envelope breaks down at the onset of mitosis. This involves the disassembly of NPCs, lamin depolymerization, and incorporation of nuclear envelope membranes into the endoplasmic reticulum ER (reviewed in Guttinger et al., 2009). Like other events in mitosis, nuclear envelope breakdown is controlled by the activity of the mitotic Cyclin/Cdk kinases. CyclinB/Cdk1 promotes NPC disassembly by phosphorylation of nucleoporins (Onischenko et al., 2005; Muhlhausser and Kutay, 2007). Peripheral Nups are the first to be dissociated from the disassembling NPCs (Terasaki et al., 2001; Dultz et al., 2008), and Nup98, the Nup involved in transcriptional memory and off-pore regulation of gene expression described earlier, is the first to be displaced (Dultz et al., 2008). Nup98 is phosphorylated at the onset of mitosis by CyclinB/Cdk1, Polo-like kinase1 (Plk1), Nek6, (and possibly other kinases) at 13 residues, most of which are localized to the C-terminal portion of the protein that mediates the interaction of Nup98 with other NPC components (Laurell et al., 2011). When these residues are mutated to sites that cannot be phosphorylated, NPC disassembly is delayed, suggesting that Nup98 phosphorylation is an initial and critical step in NPC disassembly at mitosis.

When mitosis is complete, the nuclear envelope must be re-assembled. NPCs are initially re-assembled through interactions with chromatin, followed by association of membranes to form the closed nuclear envelope. NPC re-assembly starts with the recruitment of the Nup107–160 complex to chromatin during late anaphase, mediated by the AT hook containing transcription factor 1 (AHCTF1) also known as ELYS, a scaffold nucleoporin which has a DNA binding domain for recruiting factors to chromatin (Hetzer and Wente, 2009; Imamoto and Funakoshi, 2012). Subsequently, interaction of Nup107–160 with the transmembrane Nup Pom121 allows the recruitment of membrane vesicles and also mediates interactions with other Nups (Nup93–205). Then, the central pore channel Nups and peripheral Nups are recruited to the NPCs (Guttinger et al., 2009; Capelson et al., 2010; Imamoto and Funakoshi, 2012). How are enough NPCs produced during interphase to be equally divided between daughter cells at the next mitosis? In contrast to post-mitotic NPC re-assembly, where the inactivation of mitotic Cdk1 and de-phosphorylation of Nups and other nuclear envelope proteins is required, NPC production during interphase is positively regulated by Cdk activity, in particular Cdk1 and Cdk2 (Maeshima et al., 2010). Interphase NPC assembly initiates with the entrance of the transmembrane Pom121 Nup to the nucleus, and its localization to the inner nuclear membrane (Funakoshi et al., 2011). Interestingly, in this case the ELYS Nucleoporin is not required for assembly (Doucet et al., 2010). The Nup107–160 complex is subsequently recruited, but the detailed sequence for interphase NPC assembly remains unclear (Capelson et al., 2010; Imamoto and Funakoshi, 2012).

Apart from the assembly of NPCs, their distribution in the nuclear membrane during cell cycle progression changes as well. During G1, right after completion of mitosis, NPCs are distributed unequally through nuclear surface, generating “pore-free islands” (Maeshima et al., 2006). These “pore-free islands” are rich in type A Lamins, while regions high in pore density are characterized by the presence of B-lamins and the lamin B receptor (LBR). The distribution of NPCs becomes uniform gradually as the cells progress through S and G2 phases (Maeshima et al., 2006). As NPCs and Lamins both bind chromatin and affect gene expression, the changes in distribution of the nuclear envelope proteins could potentially affect gene expression throughout the cell cycle (Figure 1).

## DNA DAMAGE AND THE NUCLEAR PORE COMPLEX

How is chromatin tethered to the nuclear pores or nuclear lamina properly replicated during S-phase? The anchoring of chromatin to NPCs turns out to have both positive and negative impacts on genome integrity during replication. For example, replication forks with persistent double strand breaks (DSBs) relocate to NPCs for repair (Nagai et al., 2008). The association of damaged forks to the pores occurs through an interaction with the Slx5/Slx8 complex, a SUMO dependent E3 Ubiquitin ligase, which is bound by Nup84 (Nagai et al., 2008; Perry et al., 2008). While it is not exactly clear why movement to the NPCs facilitates repair, it has been proposed that the nuclear periphery may provide a special permissive environment for additional DSB repair pathways beyond homologous recombination and non-homologous end joining to repair persistent DSBs (Oza et al., 2009).

While recruitment to pores can promote DNA repair, paradoxically, the anchoring of actively transcribed genes to NPCs can also be a source of replication stress. It is thought that as the DNA replication fork proceeds, it will eventually meet the NPC- tethered region actively transcribing genes. The inflexibility of tethered DNA can become a source of tension as the unwinding of DNA occurs during replication fork progression (Branzei and Foiani, 2010), and the tension generated between an actively transcribed region tethered to the NPC and the approaching replication fork is somehow released by the activity of the DNA damage checkpoint kinases and their associated complexes (Bermejo et al., 2011). When the checkpoint response is inhibited, replication forks collapse and firing of dormant replication origins occurs (Bermejo et al., 2011). It remains unclear whether a similar checkpoint mechanism is applied upon replication of transcribed genes that are not tethered to the NPC, for example those bound to other immobile nuclear structures.

The act of DNA replication during S-phase can also be a source of DNA damage (Mazouzi et al., 2014) which if not repaired could in turn lead to acquisition of mutations, cell cycle arrest or even senescence. Apart from chromatin anchoring, Nups facilitate the maintenance of genome integrity also by affecting the nuclear transport of DNA damage repair proteins required during the cell cycle. In human cells the knockdown of Nup153 impairs DNA repair by preventing proper nuclear accumulation of 53BP1 (Moudry et al., 2012). Furthermore, Tpr (Mlp1/Mlp2 in yeast), is a Nup that interacts with Nup153 in the nuclear pore basket as is also essential for proper DNA damage signaling. When Tpr is depleted, the nuclear export of p53 becomes compromised, resulting in nuclear accumulation of p53 and activation of downstream target genes such as p21 leading to premature senescence (David-Watine, 2011). Thus, NPCs influence DNA repair and DNA damage signaling during S and G2 phases in many different ways, and significantly contribute to the maintenance of genome stability.

## CELL CYCLE PHASE AND CELL FATE ACQUISITION

Cellular differentiation and proliferation must be intimately coordinated for proper development and tissue homeostasis. Stem cells pose a special case in this regard, as they must proliferate when needed, yet retain their undifferentiated status (Fuchs, 2009; Lange and Calegari, 2010; Li and Clevers, 2010). The cell cycle of pluripotent embryonic stem (ES) is reminiscent of that in early embryos, characterized by very short gap phases. Upon differentiation G1 phase becomes longer, more similar to adult somatic cells (Singh and Dalton, 2009; Calder et al., 2013; Coronado et al., 2013), and several studies have suggested ES cells initiate differentiation in G1 phase (Mummery et al., 1987; Sela et al., 2012; Chetty et al., 2013; Pauklin and Vallier, 2013). When undifferentiated human ES stem cells are isolated in different phases of the cell cycle, their propensity for spontaneous differentiation in culture varies. G1-phase cells exhibit a high rate of spontaneous differentiation, while S, and G2 -phase cells exhibit reduced spontaneous differentiation (Sela et al., 2012). Interestingly, the propensity of G1 cells to differentiate, is reduced when co-cultured with S and G2 phase cells in direct contact, suggesting cell cycle-dependent cell to cell signaling may be partly responsible for this effect. *In vivo*, the propensity for embryonic neural stem cells to self-renew vs. produce differentiated daughters also varies with changes in the cell cycle (Arai et al., 2011; Hardwick and Philpott, 2014), and manipulation of cell cycle phase length in neural stem cells can alter the balance of self-renewal vs. differentiation in the developing brain in animals ranging from *Drosophila* to mammals (Manansala et al., 2013; Tapias et al., 2014).

What are the molecular mechanisms connecting cell fate acquisition with prolonged G1? Cells in or poised to enter quiescence exhibit reduced Cyclin/Cdk activity and thus reduced phosphorylation of the Retinoblastoma (RB) tumor suppressor, a critical regulator of the restriction point and cell cycle entry (Henley and Dick, 2012; Sadasivam and Decaprio, 2013; Schachter et al., 2013). Human ES cells with hypo- or unphosphorylated RB exhibit the highest propensity to spontaneously differentiate, suggesting even a transient quiescence may consequently promote differentiation (Sela et al., 2012). However, it is important to note that a parallel study in mouse ES cells found no impact on spontaneous differentiation when Cyclin/Cdk activity was directly inhibited and RB was hypo-phosphorylated (Li et al., 2012). Whether these differences may be organism or cell-line specific remains to be determined, but multiple lines of evidence support a relationship between cell cycle changes and cell fate acquisition in human ES cells (Calder et al., 2013; Chetty et al., 2013; Coronado et al., 2013; Pauklin and Vallier, 2013; Singh et al., 2013). While the capacity for ES cells to differentiate may be established during quiescence, there is evidence that in adult cells differentiation is actively inhibited during quiescence through the transcriptional repressor Hes1 (Sang et al., 2008). Inhibition of differentiation during quiescence is critical for adult stem cells, which can spend prolonged periods in an arrested state, yet must retain their stem cell capacity (Fuchs and Chen, 2013). This suggests there will be distinct mechanisms that link the cell cycle with cell fate acquisition in adult vs. ES cells.

A view of the molecular signaling mechanisms that coordinate cell fate decisions with the core cell cycle machinery in ES cells is just beginning to emerge. Work with human ES cells has now revealed a pathway connecting CyclinD/Cdk4 activity to the TGF-β/Smad signaling pathway. TGF-β signaling promotes endoderm fate in human ES cells, but only during a permissive window in early G1. The capacity for endoderm differentiation drops-off upon cell cycle entry, in a manner correlated with increasing G1 CyclinD/Cdk4 activity. Pauklin and Vallier reconciled these observations by showing that CyclinD/Cdk4 regulates the chromatin association of the TGF-β responsive transcription factors Smad 2 and 3. Smad2/3 associate with chromatin in early G1 allowing for expression of TGF-β target genes, but CyclinD/Cdk4–dependent phosphorylation of residues in the Smad2/3 linker regions prevents them from binding chromatin upon cell cycle entry (Pauklin and Vallier, 2013). This simple relationship between CyclinD/Cdk4 activity and Smad2/3 chromatin binding creates a permissive window for endoderm differentiation directly linked to the core cell cycle machinery.

The ability to monitor differentiation and cell cycle dynamics in real-time, at the single-cell level, has been made possible by the use of the Fluorescent Ubiquitylation-based Cell-Cycle Indicator (FUCCI) system (Sakaue-Sawano et al., 2008). This system uses fluorescently labeled cell cycle reporters that are degraded at different cell cycle phase transitions, such that the dynamics of G1, S and G2/M phases can be monitored and quantified. The FUCCI system facilitated the studies of Pauklin and Vallier by allowing them to use flow cytometry to precisely sort stem cells based upon their cell cycle phase. Using a similar approach, also in human ES cells, Singh et al. (2013) examined gene expression changes during the cell cycle. They find that genes expressed specifically during G1 are heavily enriched for roles in development and cell-fate commitment and that these changes in gene expression are dependent upon cell cycle status (Singh et al., 2013). To determine how this cell cycle-dependent gene expression is regulated, they examined global chromatin changes during the cell cycle and unexpectedly found that the cytosine modification 5-hydroxymethylcytosine (5hmC) is increased during late G1, followed by a sharp decline in S-phase, and re-established during G2. Interestingly, the loss of methylation during S phase may be greater than that expected by simple passive loss through the incorporation of new unmodified nucleotides during DNA replication. If this is the case, there may be cell cycle regulated active de-methylation during S-phase in stem cells.

In contrast to the better-known repressive cytosine methylation 5mC, 5hmC is instead associated with active promoters, increased gene expression and genes poised for rapid expression (Jin et al., 2011; Pastor et al., 2011). The cell cycle regulated changes in 5hmC impact developmental gene expression and are associated with the histone PTMs H3K4me3 and H3K27me3, which are the so-called “bivalent” marks, associated with differentiation genes in stem cells. Bivalent domains have been suggested to simultaneously prevent premature expression of differentiation genes in ES cells via the repressive H3K27me3 mark, yet simultaneously keep them poised for rapid expression upon differentiation via the H3K4me3 mark, although this model is controversial (Vastenhouw and Schier, 2012; Voigt et al., 2013). The work of Singh now adds an extra layer to the puzzle by demonstrating an additional chromatin modification that appears to be under the control of the cell cycle machinery. It remains unknown how and why 5hmC is increased during the G1 phase and re-established at G2, or perhaps more importantly how and why de-methylation occurs during S phase. It will be important to investigate the molecular mechanisms linking genome methylation with the cell cycle machinery in stem cells. While it has been discussed for over two decades that the response of cells to differentiation cues seems to be affected by their cell cycle status, we are just now beginning to decipher the specific mechanisms linking the cell cycle to the chromatin state and the acquisition of cell fate.

## THE “MITOTIC ADVANTAGE” AND NUCLEAR REPROGRAMMING

While differentiation and lineage restriction of pluripotent cells seems to be increased during the G1-phase of the cell cycle, multiple lines of evidence suggest the acquisition of pluripotency or potential for nuclear reprogramming is increased during mitosis (Egli et al., 2008). An increase in nuclear reprogramming efficiency at mitosis may seem surprising at first glance, since the use of quiescent G0 nuclei was suggested to be essential to the success of the most famous example of mammalian cloning, Dolly the ewe (Campbell et al., 1996a,b). However, subsequent examples of mammalian cloning demonstrated that actively dividing cells could be efficiently used for donor nuclei (Cibelli et al., 1998). More recent cell reprogramming experiments carried out through cell-fusion of differentiated cells with mouse ES cells to form heterokaryons, suggested that successful reprogramming of chromatin actually requires activation of DNA synthesis within the first 24 h of cell fusion (Tsubouchi et al., 2013). In this case, DNA synthesis was suggested to facilitate nuclear reprogramming by passively diluting existing DNA methylation marks. But there are additional observations suggesting active cell cycling and more specifically mitosis is advantageous for nuclear reprogramming.

In studies using somatic nuclear transfer in *Xenopus*, the use of nuclei that have recently undergone mitosis was shown to increase origin accessibility in the oocyte, which poises the donor nuclei for the rapid S-phase entry and progression required during early *Xenopus* development (Lemaitre et al., 2005). Later work by Ganier et al. (2011) revealed a peculiar ability of *Xenopus* egg extracts, specifically at the metaphase stage, to increase the efficiency of reprogramming mouse fibroblast nuclei to pluripotency. Permeabilized mouse embryonic fibroblasts exposed to mitotic egg extract, but not interphase extract, exhibit decreased histone modifications such as H3K9, H3K4, and H4K20 di- and trimethylation and increased expression of pluripotency-associated genes. When somatic cell nuclear transfer was subsequently performed with the mouse fibroblast nuclei exposed to the mitotic extract, a fourfold increase in reprogramming efficiency was observed (Ganier et al., 2011). This ability of a mitotic egg extract to facilitate mammalian nuclear reprogramming was suggested at least in part, to be due to the extract promoting M-phase entry in the fibroblast nuclei. Indeed, mitotic figures and histone marks associated with mitosis were observed in the fibroblast nuclei exposed to the extract.

How exactly does the mitotic status of a donor nucleus facilitate cell fate reprogramming? Halley-Stott et al. (2014) attempted to address this question recently using a system where permeabilized adult mouse myoblast cells of different cell cycle stages are transferred into enucleated *Xenopus* oocytes, and the activation of mammalian pluripotency genes is used as a readout of reprogramming. They find, consistent with the reprogramming studies of others (Egli et al., 2008; Ganier et al., 2011), that transfer of cells with nuclei in late G2 or M-phase confers a dramatic increase in the responsiveness to reprogramming factors and induction of pluripotency genes, up to 100 times faster than that observed with interphase donor nuclei. They term this phenomenon “mitotic advantage” (Halley-Stott et al., 2014). This mitotic advantage for chromatin reprogramming to pluripotency can be observed in donor nuclei from different cell types and cannot be explained simply by the increased nuclear permeability at mitosis. The authors systematically removed different components from the mitotic chromatin to identify the molecular basis of this advantage. In sum, mitotic advantage appears to require nucleosomes, but cannot be explained by histone acetylation, phosphorylation, or methylation. Rather their data suggest that the loss of ubiquitination on histones H2A and H2B during mitosis (Joo et al., 2007) seems necessary, but is not sufficient to confer a mitotic advantage (Figure 2). Future studies will therefore be needed to identify the additional factors involved in mitotic advantage.

The work of Halley-Stott et al. (2014) suggests a permissive window for cell fate reprogramming occurs at mitosis, independent of the dilution of epigenetic marks at S-phase, acting more directly through the rapid expression of pluripotency genes. They suggest the removal of most transcription factors from mitotic chromosomes actually increases their accessibility to reprogramming factors, which allows for rapid induction upon exit from mitosis as soon as transcription resumes (Halley-Stott et al., 2014). Given the stochasticity inherent in the cellular reprogramming progress (Hanna et al., 2009), the rate of pluripotency gene induction after the completion of mitosis is likely key to successful nuclear reprogramming.

## CONCLUSIONS AND FUTURE PERSPECTIVES

Extensive connections between the cell cycle machinery and chromatin clearly exist, which impact gene expression and thus, cell fate decisions in important ways. While the use of asynchronous cell culture or mixed lineage tissues has sometimes hampered our ability to see these connections, new tools such as Chromatin Conformation Capture, the FUCCI system, the PLA and modified versions of DamID, are being used in ways that allow detailed views of the cell cycle, chromatin state and cell fate acquisition that were previously impossible. But several key questions remain unresolved. For example, does the gene expression profile of a cell, and thus cell fate, control important facets of the cell cycle such as origin choice and DNA replication timing? Or does the cell cycle status of a cell instead determine its gene expression possibilities and therefore limit choices in cell fate? If the latter is true, how can cell fate be so robustly maintained in some instances of regeneration or in cases of cell cycle disruption during development? As we learn more about the truly plastic nature of cell fate, we expect to find that the cell cycle influences the probability of acquiring certain cell fate programs, but that multiple cell cycle and cell fate states can be compatible under specific conditions. Future work will continue to uncover new molecular connections between the cell cycle machinery and developmental signaling pathways, to help us finally understand how the cell cycle impacts cell fate.

### Conflict of Interest Statement

The authors declare that the research was conducted in the absence of any commercial or financial relationships that could be construed as a potential conflict of interest.
